# Biomarkers of Endocannabinoid System Activation in Severe Obesity

**DOI:** 10.1371/journal.pone.0008792

**Published:** 2010-01-20

**Authors:** Jack C. Sipe, T. Michael Scott, Sarah Murray, Olivier Harismendy, Gabriel M. Simon, Benjamin F. Cravatt, Jill Waalen

**Affiliations:** 1 Department of Molecular and Experimental Medicine, The Scripps Research Institute, La Jolla, California, United States of America; 2 Department of Chemical Physiology, The Scripps Research Institute, La Jolla, California, United States of America; 3 Scripps Genomic Medicine-Scripps Translational Science Institute, The Scripps Research Institute, La Jolla, California, United States of America; Mayo Clinic College of Medicine, United States of America

## Abstract

**Background:**

Obesity is a worldwide epidemic, and severe obesity is a risk factor for many diseases, including diabetes, heart disease, stroke, and some cancers. Endocannabinoid system (ECS) signaling in the brain and peripheral tissues is activated in obesity and plays a role in the regulation of body weight. The main research question here was whether quantitative measurement of plasma endocannabinoids, anandamide, and related N-acylethanolamines (NAEs), combined with genotyping for mutations in fatty acid amide hydrolase (*FAAH*) would identify circulating biomarkers of ECS activation in severe obesity.

**Methodology/Principal Findings:**

Plasma samples were obtained from 96 severely obese subjects with body mass index (BMI) of ≥40 kg/m^2^, and 48 normal weight subjects with BMI of ≤26 kg/m^2^. Triple-quadrupole mass spectroscopy methods were used to measure plasma ECS analogs. Subjects were genotyped for human *FAAH* gene mutations. The principal analysis focused on the *FAAH* 385 C→A (P129T) mutation by comparing plasma ECS metabolite levels in the *FAAH* 385 minor A allele carriers versus wild-type *C/*C carriers in both groups. The main finding was significantly elevated mean plasma levels of anandamide (15.1±1.4 pmol/ml) and related NAEs in study subjects that carried the *FAAH* 385 A mutant alleles versus normal subjects (13.3±1.0 pmol/ml) with wild-type *FAAH* genotype (p = 0.04), and significance was maintained after controlling for BMI.

**Conclusions/Significance:**

Significantly increased levels of the endocannabinoid anandamide and related NAEs were found in carriers of the *FAAH* 385 A mutant alleles compared with wild-type *FAAH* controls. This evidence supports endocannabinoid system activation due to the effect of *FAAH* 385 mutant A genotype on plasma AEA and related NAE analogs. This is the first study to document that *FAAH* 385 A mutant alleles have a direct effect on elevated plasma levels of anandamide and related NAEs in humans. These biomarkers may indicate risk for severe obesity and may suggest novel ECS obesity treatment strategies.

## Introduction

Endocannabinoids (ECs) are a family of polyunsaturated fatty acid derivatives that function as lipid signaling molecules by acting as endogenous ligands at the two known cannabinoid receptors (CBRs), CB1 and CB2, present in the nervous system and peripheral organs of many living species [1]–[4]. The endocannabinoid system (ECS) is highly developed in mammals, especially in humans, and the physiology and pharmacology of the ECS have been the subject of several comprehensive reviews in recent years [5]–[7]. The most well-studied of the ECs are the fatty acid amides (FAAs) represented by N-arachidonyl ethanolamine (AEA) or anandamide, N-palmitoyl ethanolamine (PEA), N-oleoyl ethanolamine (OEA) and related N-acylethanolamine (NAE) derivatives together with the esters of arachidonic acid including 2-arachidonyl glycerol (2-AG) as well as other lipid signaling molecules acting at CBRs [2], [7]–[10]. Some of these ECs are known to modulate a variety of physiological functions including synaptic transmission, immune function, nociception, fertility, and cardiovascular function, in addition to nervous system development, and are involved in many pathophysiological disease processes [5], [7], [11].

The complex biosynthetic and enzymatic degradation pathways for these ECS signaling molecules are currently being investigated as potentially selective disease treatment targets [6], [11], particularly fatty acid amide hydrolase *(FAAH)* and monoglyceride lipase *(MGLL)*, which are the principal inactivating enzymes for FAAs and 2-AG. There is an increasing body of data on endocannabinoid control of energy homeostasis and recent evidence for ECS dysregulation in overweight and obesity disorders [12]–[17]. It has become apparent that human obesity is associated with activation of the ECS and with increased levels of ECs in many tissues, as well as in the circulation [17] However, the underlying molecular mechanisms of ECS dysregulation in obesity need clarification to better understand ECS risk factors in obesity and to develop novel selective treatment strategies.

Current concepts in this field indicate that body weight and fat metabolism are influenced by complex ECS regulatory mechanisms that modulate energy balance, feeding behavior and peripheral lipid metabolism [18], [19]. The ECS comprises one of the most important weight regulatory systems in humans, since endocannabinoids have numerous effects on energy homeostasis by activating cannabinoid receptors throughout the nervous system and in the periphery [8] together with modulation of other downstream energy regulating molecules. In the mammalian brain, ECs modulate appetite via leptin-regulated circuits in the lateral hypothalamus or by direct activation of the mesolimbic dopamine “reward” pathways for food intake and thus may increase feeding behavior in animal models of obesity [19]. In mammalian peripheral tissues, ECs may have direct effects, for example in adipose tissue where CB1 receptors are located and ECS biosynthetic and degradative enzymes are expressed [20], [21], in the liver where ECs promote hepatic lipogenesis [12], in the intestine where OEA [22], the monounsaturated analogue of anandamide has the opposite or anorexic effect of decreasing food intake and body weight in animal models [15], as well as ECS effects in other tissues such as skeletal muscle [16] and endocrine pancreas [23].

Recent investigations have demonstrated activation of the peripheral ECS in human obesity and type II diabetes [24] as well as increased circulating plasma AEA levels in young women with binge eating disorder [25]. Increased plasma AEA and 2-AG levels have been found in obese menopausal women compared to lean women of similar age [26]. A study in a human visceral obesity disorder in men has shown elevated plasma fasting levels of 2-AG but not AEA as measured by liquid chromatography-mass spectroscopy [27]. Also, a strong negative correlation has been found between *FAAH* expression in adipose tissue and circulating ECs [26]. Other studies of plasma AEA and 2-AG levels have shown no relationship with obesity or body fat distribution [17], but these findings may be related to differences in methodology or obesity phenotypes in the study cohorts. Importantly, the pathophysiological role of circulating endocannabinoids as risk factors for obesity remains to be elucidated.

The overall aim of the present study was to investigate circulating biomarkers of ECS activation in severely obese subjects compared to normal weight controls using highly sensitive triple-quadrupole mass spectroscopy plasma quantification methods, combined with genotyping for common mutations in the genes encoding the endocannabinoid and fatty acid amide inactivating enzymes *FAAH* and *MGLL*. This aim is based on our previous reports that showed an association of the common human *FAAH* 385 C→A (P129T)non-synonymous mutation with overweight and obesity [28] and biochemical evidence of approximately 50% reduced cellular expression and activity of mutant human *P129T FAAH* [29]. This study achieved the aim of identifying both plasma and *FAAH gene* ECS biomarkers of severe obesity that have the potential to be clinically useful if confirmed by larger studies.

## Methods

### Objectives

The objective of this study was to investigate readily available circulating biomarkers of ECS activation in severely obese subjects compared to normal weight subjects by combining accurate measurement of plasma endocannabinoid levels with SNP genotyping in critical ECS inactivating enzymes. The main hypothesis herein based on previous studies was that plasma levels of anandamide and related NAEs would be elevated in severely obese carriers of the *FAAH* 385 A alleles compared to normal weight wild-type *FAAH* carriers. This evidence could further support ECS activation in a sub-type of severely obese individuals and may indicate novel ECS treatment targets in severe obesity.

### Participants

Samples and linked biometric data used in this study were obtained from the rimonabant (Acomplia) CRESCENDO study (http://clinicaltrials.gov/ct/show/NCT00263042), a cardiovascular outcomes trial to assess the efficacy of the CB1 receptor antagonist, rimonabant (Acomplia), in reducing the risk of heart attack, stroke, or cardiovascular death in abdominally obese individuals and comprised 18,671 randomized and treated subjects and was conducted by Sanofi-Aventis. Subjects and samples for this study were from a subset of 3101 subjects who volunteered with separate written informed consent for the CRESCENDO Genomic Substudy cohort. A subset of the substudy were selected for this ECS study based on BMI (150 with BMI<30 and 150 with BMI>40). Severely obese subjects for this ECS metabolite and genomics study were selected from the Genomic Substudy cohort based on a body mass index (BMI) of 40 kg/m^2^ to 60 kg/m^2^ and waist circumference of greater or equal to 140 cm in men and 131 cm in women. Normal weight controls were included based on a BMI of less than 26 kg/m^2^ and a waist circumference of less than or equal to 100 cm in men and 90 cm in women. To reduce false-positive findings due to differing genetic backgrounds, and since Whites were highly represented in the patient population sampled, the study was limited to male and female Whites, ranging in age from 55 to 77. All study participants and data were anonymous and biometric data were linked to each plasma and DNA sample by unique study code (see Dataset S1). The anonymous biometric data and the subjects' samples were obtained with broad informed consent approved by the Institutional Review Board (IRB)/EC for the site and use of anonymous samples in the current study received additional approval from the Scripps IRB.

### Procedures: Plasma Endocannabinoid and NAE Analysis

Whole blood samples were collected in evacuated glass tubes containing EDTA. Samples were centrifuged to separate plasma from blood cells and plasma was withdrawn and stored in 1 ml aliquots at −80°C prior to plasma lipid extraction. For each sample, 0.5 ml plasma was added to a glass vial containing 2.0 ml chloroform (CHCl3), 1.0 ml methanol (MeOH) and 0.5 ml (1% v/v) formic acid. To this mixture were added aliquots of 10 pmol D5-2-arachidonlyglycerol (2-AG) and 5 pmol D8-arachidonlyethanolamine (AEA). Vial contents were vortex mixed for 30 seconds and centrifuged at 10°C (1400×g for 10 minutes). The organic layer was carefully removed avoiding the aqueous layer and dried under a stream of nitrogen (N_2_) gas. The lipid layer was then re-solubilized in 100 microliters of 2∶1 CHCl_3_∶CH_3_OH.

Quantitative analysis of endocannabinoids and NAE analogues using the deuterated standards for AEA and 2-AG together with calculation of the other EC analogues was based on a ratio to the deuterated standards [30] and was performed on the Agilent 6410 Triple Quadrupole Liquid Chromatography-Mass Spectroscopy (TQMS) instrument using positive ion analysis mode. For each sample, 20 microliters of re-solubilized plasma lipids were injected into the TQMS instrument and EC congeners were measured by multiple reaction monitoring (MRM) using the following transitions: 348>62 (AEA), fragmentation energy = 8, and 379>287 (2-AG), fragmentation energy = 11. Chromatography was performed using the following solvents: **A** - 95∶5∶0.1 H20∶methanol∶formic acid and **B** – 60∶35∶5∶0.1 isopropanol∶methanol∶H2O∶formic acid. Lipids were injected into a 5 microcentimeter C^18^ column (50×4.6 mm) from Phenomenex (Torrance, CA) and eluted with a 10 minute solvent B gradient from 60% to 100%. Values for each endocannabinoid and NAE analogue were subsequently calculated using ratios to the deuterated internal standards to calculate absolute concentrations, expressed as picomoles (pmol) of EC metabolite per ml of plasma similar to current methods [30].

### Procedures: FAAH and MAGL SNP Genotyping Methods and Study Subjects

Genotyping of the SNPs in the *FAAH* (GeneID 2166) and *MGLL* (GeneID 11343) from dbSNP was performed using the Sequenom platform. The major allele frequencies were determined in all 300 obese and normal subjects, using the Sequenom data for detection of 4 common SNPs in *FAAH* and 16 in *MGLL* (data not shown). To reduce false-positive findings due to differing genetic backgrounds, the same patient population used for the EC metabolite analysis was sampled and only male and female Whites were included, as noted above.

### Procedures: Statistical Methods

The *apriori* or pre-study power calculation to detect a true association in this study was based upon estimations from data reported for measurement of anandamide levels in humans in a clinical trial [31]. Using a standard deviation (SD) for anandamide of approximately 4 pmoles/ml, the power was 0.9 to detect a difference of 2.2 pmoles/ml (standardized difference 0.53) and power of 0.8 to detect a difference of 1.8 pmoles/ml (standardized difference 0.45) at alpha = 0.05 with an expected total sample size of 150.

The distribution of each metabolite was assessed for outliers and normality. Log transformation of data resulted in normal distributions for all EC analogues. Stratification revealed no significant differences in mean metabolite levels by age and sex and these variables were thus not included in further analyses. Geometric means of each metabolite were compared between BMI groups using the t-test. Frequencies of the *FAAH* genotype were compared between the severely obese and normal weight groups using the Fisher's exact test. Geometric mean levels of EC analogues were compared between *FAAH* genotype groups (carriers of the minor “A” allele compared with subjects with the wild type genotype C/C) in both BMI groups combined using the t-test and stratified by BMI group using 2-Way ANOVA. Normal weight wild-type *FAAH* subjects were compared with severely obese carriers of the FAAH 385 mutant A allele using 1-Way ANOVA with Dunnett's Correction for multiple comparisons. A two-sided p-value <0.05 was considered statistically significant.

### Ethics

Participants in this study initially gave broad written informed consent for collection and analysis of plasma and DNA for the Sanofi-Aventis CRESCENDO Genomic Substudy. In this study of EC analogues, all subjects were anonymous and identified only by a code number linked to biometric data. Because informed consent from strictly anonymous CRESCENDO study subjects was not possible, the present study received an exemption for additional written consent from the Scripps IRB.

## Results

### Endocannabinoid and NAE Levels

Demographic characteristics of the 96 severely obese subjects and 48 normal weight controls included in the study are shown in Table 1. The severely obese group had a mean age that was approximately 5 years younger than the normal weight group (p<0.0001). Other variables including the percentage of male subjects (44% in severely obese vs. 35% in the normal weight group) were not statistically different.

**Table 1:** Demographic Characteristics of EC Metabolite Study Subjects.

Characteristic	Severely Obese (n = 96)		Normal Weight (n = 48)	
	**Mean (SD)**	**Range**	**Mean (SD)**	**Range**
**Age (years)**	61.9 (5.8)	55–77	66.4 (6.7)	55–77
**Body Mass Index (kg/m^2^)**	49.2 (3.2)	45–58	24.7 (1.2)	22–26
	**n**	**%**	**N**	**%**
**Male**	43	44.8	17	35.4
	**Mean (SD)**	**Range**	**Mean (SD)**	**Range**
**Waist circumference (cm), Males**	151.2 (8.1)	140–170	103.3 (0.4)	102–104
**Waist circumference (cm), Females**	140.7 (7.0)	131–157	93.7 (3.3)	89–100

The baseline mean endocannabinoid and NAE levels (Table 2) were not significantly different among the subjects in the two BMI groups except for high LEA (p = 0.04) and low DHEA (p = 0.002) plasma levels. There was a trend to elevated anandamide (AEA) levels (p = 0.06) in severely obese subjects that was not quite significant (Table 2).

**Table 2:** Mean plasma ECS analogue levels (picomoles/ml) by body mass index (BMI).

Metabolite	Severely Obese n = 96	Normal Weight n = 48	p**
	Mean* pmol/ml (95% CI)	Mean* pmol/ml (95% CI)	
**AEA Anandamide arachidonylethanolamide**	14.5 (13.6, 15.6)	12.8 (11.4, 14.5)	0.06
**2AG 2-arachidonylglycerol**	5.4 (4.8, 6.1)	5.6 (4.6, 6.8)	0.73
**C22:6 DHEA Docosahexaenoylethanolamine**	7.5 (7.0, 8.1)	9.2 (8.3, 10.1)	0.002
**C16:0 PEA Palmitoylethanolamine**	215.3 (203.8, 227.4)	213.3 (190.1, 239.3)	0.87
**C18:0 SEA Stearoylethanolamine**	76.6 (71.4, 82.1)	80.1 (70.5, 92.7)	0.44
**C18:1 OEA Oleoylethanolamine**	118.6 (111.2, 126.6)	107.9 (97.2, 119.8)	0.11
**C18:2 LEA Linoleoylethanolamine**	44.7 (41.5, 48.0)	38.9 (34.9, 43.4)	0.04

**CI = confidence interval.**

*geometric mean based on log-transformed data.

**Student's t-test.

### SNP Genotyping

Analysis of the *FAAH* and *MGLL* genes for common gene variations or SNPs was completed and there were no common variants of *MGLL* found in this study. The only coding variant commonly found in the subjects in this obesity study was the *FAAH* 385 C→A (P129T) as previously reported [28]. In the ECS metabolite analysis group (n = 144) frequency of the mutant A/A genotype was 7.3% in the severely obese compared to 4.2% A/A in the normal weight group, a nearly 2 fold difference similar to the previous study [28] that was not statistically significant here, probably because this study was not powered to detect a significant genotype difference.

### Plasma ECS Analogue Levels Correlated with the *FAAH* P129T Mutation

**Figure 1:**
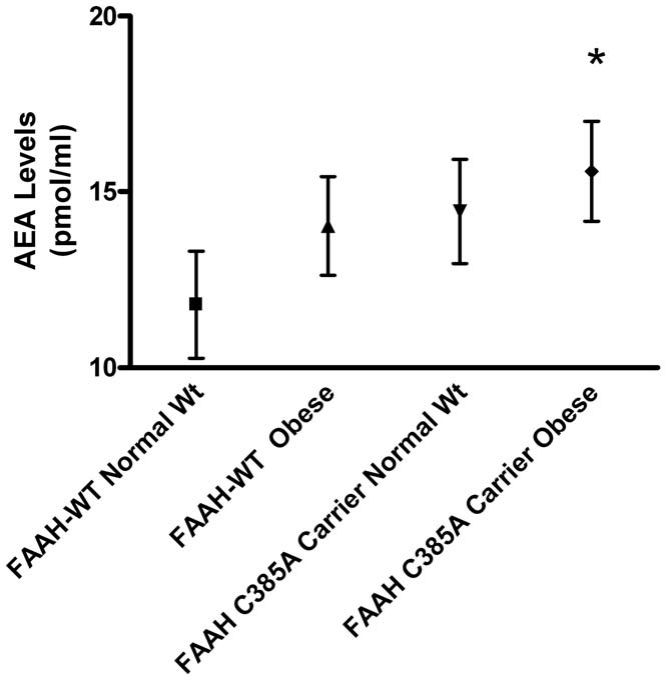
Geometric mean Anandamide (AEA) levels by BMI group and *FAAH* genotype. Error bars indicate 95% confidence intervals for geometric means. *p<0.05 compared with *FAAH* wildtype normal weight controls using 1-way ANOVA with Dunnett's correction for multiple comparisons but p>0.05 for all other pairwise comparisons.

To evaluate the effects of the *FAAH* C385A mutation on the circulating plasma levels of endocannabinoids and NAE analogues, geometric mean congener levels were compared between carriers of the *FAAH* 385 mutant A allele and homozygotes for the wild type (WT) C alleles Using the t-test comparing geometric mean plasma metabolite levels in the two groups, there was a significant elevation of mean anandamide (AEA) levels (p = 0.04) and related NAE levels in carriers of the *FAAH* 385 mutant A alleles versus *FAAH-*WT carriers (Table 3.). An analysis comparing severely obese *FAAH 385* A carriers versus normal weight *FAAH-*WT subject groups using 2-way ANOVA which controls for BMI to analyze the direct effect of *FAAH* 385 A mutant alleles compared to subjects with the wild-type *FAAH* C/C genotype also showed significant (p<0.05)) elevation of AEA and related NAEs (Table 4). In the 2-way ANOVA analysis (Table 4), the interaction terms (genotype X BMI category) were not statistically significant for any of the EC analogues listed. The findings were internally consistent and statistically significant using multiple statistical methods as noted. As shown in Figure 1, comparing the four subject groups including *FAAH-*WT normal weight, *FAAH-*WT Obese, *FAAH* C→A normal weight and *FAAH* C→A Obese, revealed a trend toward increasing mean AEA levels with significantly elevated AEA (p<0.05) in obese *FAAH* mutant A allele carriers versus *FAAH-WT* normal weight subjects using 1-Way ANOVA with Dunnett's correction for multiple comparisons. This amounts to a modest but significant 1.14 fold higher mean AEA plasma level in severely obese mutant *FAAH* carriers compared to normal *FAAH* controls. Several other N-acylethanolamines related to AEA that are metabolized by *FAAH*, including PEA, OEA and LEA, were also significantly elevated in severely obese mutant carriers versus normal controls (see Dataset S1). Plasma levels of the endocannabinoid 2-AG, which is not metabolized by *FAAH*, showed no difference comparing severely obese and normal subjects (Tables 3 and 4).

**Table 3:** T-test Comparison between *FAAH C385A* carriers and wild-type subjects with BMI groups combined.

Metabolite	FAAH C385A (P129T)Genotype	Geometric Mean (95%) CI	p-value
**AEA**	C/C	13.3 (12.3, 14.4)	0.04
	A/C + A/A	15.1 (13.7, 16.8)	
**2-AG**	C/C	5.5 (4.8, 6.3)	0.97
	A/C + A/A	5.5 (4.7, 6.4)	
**C22:6 DHEA**	C/C	7.8 (7.2, 8.3)	0.09
	A/C + A/A	8.6 (7.7, 9.6)	
**C16:0 PEA**	C/C	198.7 (189.7, 208.1)	0.0006
	A/C + A/A	229.8 (213.3, 247.5)	
**C18:0 SEA**	C/C	71.7 (67.5, 76.1)	0.004
	A/C + A/A	83.6 (76.3, 91.6)	
**C18:1 OEA**	C/C	108.0 (100.5, 115.9)	0.003
	A/C + A/A	128.0 (118.0, 138.9)	
**C18:2 LEA**	C/C	40.9 (37.9, 44.2)	0.08
	A/C + A/A	45.8 (41.4, 50.6)	

**Table 4:** Comparison of Mean Plasma ECS Analogue Levels by FAAH 385 Genotype Controlling for BMI.

		BMI Group		
Metabolite	FAAH (385) P129TGenotype	Low BMI	High BMI	p-value for A carriers vs. wildtype controlling for BMI group (2-Way ANOVA)
**AEA**	C/C	11.8 (10.0, 13.9)	14.0 (12.9, 15.3)	0.04
	A/C + A/A	14.4 (12.0, 17.4)	15.6 (13.7, 17.7)	
**2-AG**	C/C	5.4 (4.0, 7.3)	5.5 (4.7, 6.4)	0.94
	A/C + A/A	5.9 (4.5, 7.6)	5.2 (4.3, 6.4)	
**C22:6 DHEA**	C/C	9.0 (7.9, 10.1)	7.2 (6.6, 7.8)	0.08
	A/C + A/A	9.4 (8.0, 11.2)	8.1 (7.0, 9.4)	
**C16:0 PEA**	C/C	195.0 (173.5, 219.1)	200.3 (191.4, 209.6)	0.005
	A/C + A/A	220.3 (191.3, 253.7)	235.8 (216.0, 257.7)	
**C18:0 SEA**	C/C	75.0 (65.8, 85.5)	70.3 (65.7, 75.2)	0.02
	A/C + A/A	80.0 (67.6, 94.8)	85.9 (76.9, 95.9)	
**C18:1 OEA**	C/C	96.5 (83.7, 111.3)	113.4 (104.6, 123.0)	0.003
	A/C + A/A	126.2 (110.2, 144.5)	129.1 (116.0, 143.7)	
**C18:2 LEA**	C/C	37.4 (32.1, 43.4)	42.6 (39.0, 46.6)	0.08
	A/C + A/A	41.2 (34.9, 48.7)	48.8 (43.0, 55.4)	

## Discussion

The principal new findings of this study were that subjects with the *FAAH* 385 A mutant alleles controlling for BMI had modest but significant elevation (p = 0.04) of AEA and related NAEs (Table 3; Figure 1) There was also modest but significantly elevated (p<0.05) circulating mean plasma levels of anandamide (AEA) in severely obese carriers of the *FAAH* 385 mutant A alleles compared to normal BMI subjects with the wild-type *FAAH* genotype Table 4; Figure 1). In Figure 1, there is the appearance of elevated AEA levels in several groups but only the comparison of the control *FAAH-*WT normal weight subjects with the *FAAH 385 A* carrier obese was statistically significant (p<0.05). Because plasma levels of N-acylethanolamine analogues related to AEA, including PEA, OEA and LEA, are significantly elevated in severely obese carriers of the mutant *FAAH* 385 A alleles, this supports the concept of a functional *FAAH* enzyme mutation in subjects with *FAAH* 385 C→A SNPs. Taken together, these findings provide further evidence of activation of the ECS in severely obese subjects with the *FAAH* 385 C→A mutation. Despite the relatively small subject cohort, tight plasma ranges and statistical significance (Figure 1) was achieved by using highly sensitive triple quadrupole mass spectroscopy methods. All statistical methods using both the original dataset and log-transformed dataset confirmed the internal and external validity of this study. These findings are statistically robust with significant two-sided p values ranging from p = 0.04 to p<0.05 in the case of AEA to p = 0.003 in the case of OEA (Tables 4 and Figure 1). Although there is a modest 1.14 fold elevation of mean plasma AEA levels in severely obese mutant *FAAH* carriers, the findings are nevertheless statistically significant in this careful case-controlled study.

These findings are noteworthy because endocannabinoid signaling molecules like AEA are known to regulate food intake and fat metabolism in humans. The role of endocannabinoid system dysregulation as a risk factor in the development of obesity has been the subject of considerable recent interest [13], [15], [17], [26]. Activation of the ECS in obesity [17] has the potential to identify clinically useful biomarkers of ECS risk factors that may contribute to severe obesity through appetite stimulation, high fat intake and fat metabolism.

In the present study, we present the first evidence of a direct effect of *FAAH* 385 A mutant alleles on elevated mean basal levels of anandamide (AEA) in carriers of the *FAAH* P129T mutation. This is substantiated by demonstration of several of elevated N-acylethanolamine analogues related to AEA that are also metabolized by *FAAH*. In this study, the *FAAH* 385 C→A (P129T) mutant genotype, which is the principal catabolic enzyme of many N-acyl ethanolamine ECS signaling molecules, has the same approximately 2-fold prevalence (7.3% vs. 4%) in severely obese subjects compared to normal BMI subjects observed in a previous study (28). Among both obese and normals, carriers of the *FAAH* 385 minor A alleles had higher levels of AEA and other NAEs, except 2-AG which is not metabolized by FAAH. Likewise, all endocannabinoid levels were positively correlated with each other except 2-AG (Table 4). Of particular interest is the significant (p<0.05) but modest elevation of AEA in subjects with the *FAAH* 385 mutant A alleles (Figure 1), since AEA activates the CB1 receptor and has the potential to stimulate appetite and feeding behavior as well as modulate several metabolic functions including fatty acid metabolism and contributes to diet-induced obesity [12], [16].

At the present time, there are four human clinical studies investigating the association of the functional *FAAH* P129T mutation with obesity. In two larger studies, one demonstrated a significant association of the *FAAH* P129T genotype variant with overweight and obesity [28] and one study showed no significant association of this variant with fat accumulation phenotypes [32]. In two smaller clinical studies, one confirmed the significant over-representation of the *FAAH* 385 A alleles in overweight/obese subjects [33] and one study comparing healthy and obese subjects with metabolic syndrome found no significant association with the *FAAH* P129T variant after correction for multiple comparisons [34]. Thus, more clinical studies with comparable subject cohorts are needed to evaluate the significance of the association of the *FAAH* 385 A mutant alleles with overweight and obesity. Since the relatively common *FAAH* P129T mutation has been associated with obesity in some studies [28], [33], it is possible that other rare *FAAH* variants or *MGLL* variants may also contribute to ECS tonic activation in severely obese individuals. These significant AEA elevations suggest that mean basal plasma ECS metabolite levels measured with sensitive techniques and stratified by metabolizing *FAAH* enzyme genotype may be a reliable indicator of biologic dysregulation in the endocannabinoid system.

This study is the first to document that the *FAAH* 385 A mutant alleles directly affect elevated plasma levels of AEA and related NAEs and suggests that using biomarkers including AEA, PEA, OEA and LEA plasma levels combined with genotyping for the minor *FAAH* 385 variant A allele may identify a new ECS-related obesity phenotype with a higher risk for severe obesity. Larger clinical obesity studies of plasma endocannabinoid and NAE levels correlated with *FAAH* P129T genotype are needed in normal, mildly overweight and severely obese subjects to confirm the presence of linked plasma and *FAAH* gene ECS biomarkers for the risk of severe obesity since independent confirmation could, in turn, lead to more selective endocannabinoid or EC analogue treatment strategies.

## Supporting Information

Dataset S1 (0.07 MB XLS)
